# Gene expression in bumble bee larvae differs qualitatively between high and low concentration imidacloprid exposure levels

**DOI:** 10.1038/s41598-023-36232-y

**Published:** 2023-06-09

**Authors:** Rubén Martín-Blázquez, Austin C. Calhoun, Ben M. Sadd, Sydney A. Cameron

**Affiliations:** 1 Department of Entomology, University of Illinois Urbana-Champaign, Urbana, IL USA; 2 School of Biological Sciences, Illinois State University, Normal, IL USA; 3 Present Address: Department of Evolutionary Ecology, Estación Biológica de Doñana, Consejo Superior de Investigaciones Científicas (CSIC), Isla de la Cartuja, Seville, Spain

**Keywords:** Animal behaviour, Functional genomics, Gene expression, RNA sequencing

## Abstract

Neonicotinoid pesticides negatively impact bumble bee health, even at sublethal concentrations. Responses to the neonicotinoid imidacloprid have been studied largely at individual adult and colony levels, focusing mostly on behavioral and physiological effects. Data from developing larvae, whose health is critical for colony success, are deficient, particularly at the molecular level where transcriptomes can reveal disruption of fundamental biological pathways. We investigated gene expression of *Bombus impatiens* larvae exposed through food provisions to two field-realistic imidacloprid concentrations (0.7 and 7.0 ppb). We hypothesized both concentrations would alter gene expression, but the higher concentration would have greater qualitative and quantitative effects. We found 678 genes differentially expressed under both imidacloprid exposures relative to controls, including mitochondrial activity, development, and DNA replication genes. However, more genes were differentially expressed with higher imidacloprid exposure; uniquely differentially expressed genes included starvation response and cuticle genes. The former may partially result from reduced pollen use, monitored to verify food provision use and provide additional context to results. A smaller differentially expressed set only in lower concentration larvae, included neural development and cell growth genes. Our findings show varying molecular consequences under different field-realistic neonicotinoid concentrations, and that even low concentrations may affect fundamental biological processes.

## Introduction

The decline of bumble bee (*Bombus*) species globally has become a major recent concern^1^, with many surveys in Europe^2,3^, North America^4–6^ and South America^7,8^ showing species reductions in distribution and relative abundance, some threatened with extinction. This decline of bumble bee populations has the potential to significantly reduce wild plant and agricultural crop pollination^9–11^, making it critical to understand potential threats. Multiple causes of these declines have been proposed^1,12^, including climate change^13–15^, changes in land use^16–18^, nutritional stress^19–21^, and exposure to pathogens^22–24^ and pesticides^25–27^. Assessing how these stressors may negatively affect the health of bumble bee individuals and colonies is paramount to identify the underlying drivers of declines and to moderating the threats to bumble bee populations globally^1^. To do this, behavioral, physiological, and molecular responses to stressors must be assessed at relevant life stages of development. We use a transcriptomic approach to uncover gene expression differences upon exposure to two different field-realistic concentrations of the neonicotinoid pesticide imidacloprid in the relatively understudied larval stage of *Bombus impatiens.*

Neonicotinoid pesticides have been used widely for agricultural pest control in recent decades^28^. A major increase in their use has occurred during the decades of observed bumble bee declines, establishing them as a potential major threat to bumble bee health^1,29,30^. As systemic pesticides predominantly applied as seed coatings, neonicotinoids accumulate in all plant tissues during growth and development^31^, including the nectar and pollen^32^. As a result, beneficial non-target insects such as pollinators can be exposed to their harmful effects^32^. Although field concentrations of some neonicotinoids, including imidacloprid, are usually lower than the lethal oral dose (LD_50_) determined for bumble bees (20–40 ng per bee)^33^, ongoing exposure to lower concentrations (0.7–51 ppb^34^) cause protracted sublethal effects^21,35–40^. Such effects include reduction of foraging efficiency^41,42^, learning and short-term memory impairments^43^, disruption of immune response^44^, reduction of queen hibernation success^45^, colony initiation and development^21,46–49^ and reproduction^49,50^.

The majority of studies on the effects of neonicotinoid exposure on bumble bees have been performed at the colony, sub-colony (microcolony) or individual adult levels. Effects on bumble bee larvae, however, remain understudied^51^, even though it has been recommended that effects on larval development should be included in pesticide risk assessment studies^52,53^. In fact, expected probabilities of pesticide exposure through the routes of wax residues, nectar and pollen are considered to be as high or higher in bumble bee larvae relative to adults^54^. In previous studies of other bee species, larval exposure to pesticides including neonicotinoids has resulted in detrimental effects on both larval and emerged adult survival^55–58^. In the solitary bee species *Osmia cornuta* and *O. bicornis*, larval exposure to the neonicotinoid thiacloprid increased developmental mortality and development time, and decreased pollen provision consumption and cocoon weight^59^. In addition to the effects that are apparent during larval development, exposure of larvae to neonicotinoids can have subsequent negative effects on adult bee traits, including morphology^55^ and olfactory learning^60^. For instance, exposure of honey bee larvae to imidacloprid reduces microglomerular density in the mushroom bodies, resulting in olfactory-associated behavioral impairment in adults^61–63^. Similarly, larval exposure of *B. impatiens* to sublethal concentrations of Spinosad, a biopesticide that acts on the same receptors as neonicotinoids, negatively affected foraging efficiency in adults^64^.

Analyzing the molecular responses of bumble bee larvae to different concentrations of neonicotinoids within field-realistic ranges is an important endeavor that will add to our understanding of the full range of effects that these pesticides can have on bumble bee health. Whole genome transcriptome analysis (RNA-seq) has a great utility when approaching questions of bee health, by improving our understanding of the associations between molecular, physiological and behavioral responses to stressors such as pesticides^65^. A transcriptomic study of the effects of exposure to the neonicotinoid thiamethoxam on honey bees illustrates how effects on fundamental biological pathways, here including pentose phosphate pathways, starch and sugar metabolism and sulfur metabolism, can be uncovered, which would remain hidden in other, whole organism level approaches^66^. This can include the discovery of potentially interconnected effects on critical pathways, such as the influence of pesticide exposure on honey bee immunity^67^, or indicators of adverse long-term effects^65^. Furthermore, the identification of conserved pathways involved in pesticide exposure responses can lead to their potential implementation as biomarkers for assessment in colonies and populations^65^.

RNA-seq studies have shown that exposure to sublethal neonicotinoid concentrations can affect gene expression in honey bees and bumble bees^68–74^. However, molecular responses to exposure may vary depending on the context. For instance, research on *B. terrestris* found that clothianidin had a greater impact than imidacloprid on gene expression in head tissue, and the impact was greater in workers than in queens^70^. Furthermore, the neonicotinoid clothianidin affected the expression of detoxification genes in a sex-specific manner in *B. impatiens*^73^. Comparing across these studies is difficult, because each of them used chronic exposures differing in both duration and dose. Additionally, each of these studies used a single sublethal concentration that is in the mid-high range of reported field-realistic concentrations (see Supplementary Table S1 for details). This also highlights the need for transcriptome approaches in bumble bees that compare responses to different sublethal field-realistic concentrations.

The goal of our research is to characterize differences in larval gene expression associated with exposure to two different sublethal concentrations of imidacloprid (0.7 and 7.0 ppb). These concentrations have been shown previously to have differential effects on adult bumble bee immunity^44^. Larvae were exposed in microcolonies through spiked provisions of pollen and sugar water. We hypothesize that exposure of developing larvae to both concentrations will alter gene expression in pathways associated with fundamental biological processes, but that the higher concentration of imidacloprid will have greater qualitative and quantitative effects on the larval gene expression profiles. We predict that the expression of genes related to the response to stress and nervous system development will be affected by both concentrations, but that larvae exposed to the higher imidacloprid concentration will exhibit a greater number of differentially expressed genes in these categories than those exposed to the lower concentrations. To verify the use of imidacloprid-spiked food provisions and to provide additional context to interpret the gene expression results, we recorded the total use of the treatment provisions during the period of exposure. This is important to consider because an alteration of larval feeding by nursing workers and food consumption of larvae themselves has been shown in *Apis*^60^ and *Osmia*^59^, respectively, when exposed to neonicotinoids. This study sheds light on the effects of imidacloprid at the molecular level in bumble bee larvae, providing a pathway to understanding some of the mechanisms that could lead to harmful impacts during this at risk but under-studied stage, which could have cascading effects on adult, and ultimately colony, health.

## Materials and methods

### Bumble bee source colonies

Wild queens of *B. impatiens* were collected upon emergence from hibernation from a conservation-easement natural area in the Mackinaw River watershed (Lexington, IL, USA) on 26–30 April 2018 with the permission of the ParkLands Foundation (http://www.parklandsfoundation.org). Colonies were reared under red-light at 26 °C (± 1.5 °C) and 50% relative humidity following the methods described in^75^. Briefly, they were fed inverted sugar water (1 g cane sugar, 1 ml boiled water, 0.1% cream of tartar) ad libitum and honey bee pollen (Brushy Mountain Bee Farms, Moravian Falls, NC, USA) three times per week. Following microcolony establishment (see below), honey bee-collected pollen (CC Pollen Co., https://www.beepollen.com, Phoenix, AZ, USA), gathered in high desert habitat away from agricultural or residential areas and deemed pesticide-free^76^, was provided.

### Microcolony design and imidacloprid treatments

Four laboratory-reared *B. impatiens* colonies (C01, C02, C03 and C04) served as sources for the microcolonies. From each source colony, an individual microcolony was established for each of three imidacloprid treatments: control, 0.7 ppb imidacloprid and 7.0 ppb imidacloprid (total number of microcolonies: n = 12). Each microcolony comprised five workers and a brood clump with seven (mean ± SE = 7.000 ± 0.103) size-controlled larvae in a plastic box (17 cm L × 12 cm W × 10 cm H). A larval size that approximated to third instar larvae was chosen, with larval size/instar determined by visual comparison with other instars of smaller (younger first and second instars) or larger (older prepupal larvae) sizes. Microcolonies were provisioned ad libitum with sugar water and pollen dough in a small petri dish. Initial provisions were untreated pollen and sugar water, allowing microcolonies to acclimate for 48 h after establishment. After acclimation, each microcolony was given its respective imidacloprid treatment provision (control, 0.7 ppb imidacloprid and 7.0 ppb imidacloprid; see the section below). 48 h after imidacloprid treatment initiation, three larvae per microcolony were flash frozen in liquid nitrogen and stored at − 80 °C for later RNA-seq analysis. A 48 h imidacloprid exposure period was chosen because previous studies of bees have shown significant gene expression changes at this time in response to neonicotinoids^77^ and other insecticides^78^.

### Imidacloprid concentrations and preparation

Imidacloprid was provided to microcolonies at 0.7 ppb (low) and 7.0 ppb (high) concentrations through provisioned sugar water and pollen dough. The concentrations were chosen based on reported concentrations that bumble bees are often exposed to in the field^44^. Concentrations up to 1000 ppb have been detected in pollen and nectar^23^, but levels between < 1 and 15 ppb are typical^36,37,79,80^. Imidacloprid (Millipore Sigma, 37,894) stock solutions (10,000 ppb) were prepared in ultrapure water, and diluted immediately prior to use. Pollen dough was made by mixing sugar water and ground honey bee pollen at a ratio of 1:3.2 (v/w). Controls comprising untreated sugar water and pollen provisions were prepared in the same way, but without the addition of imidacloprid.

### Microcolony use of pollen and sugar water provisions

To ensure the use by microcolonies of imidacloprid-spiked resources and to potentially provide context for any differences in gene expression outcomes, sugar water and pollen use during the experimental imidacloprid exposure period was monitored. Sugar water use per microcolony was measured as the sugar water volume difference between the start and the end of the 48 h treatment period. All pollen dough remnants from each microcolony were dried at 55 °C for 48 h and weighed individually. Because dry weight of each pollen pellet could not be assessed prior to provisioning, pollen use was estimated as the mass difference between the dried pollen remnant and the mean dry weight of 10 consistently and identically made intact pollen dough provision standards. This approach has been used to approximate consumption of resources in other microcolony studies^75^. Both sugar water and pollen use were standardized by the number of days of the treatment and by the number of bumble bee adults and larvae in each microcolony. Due to the experimental design and distribution of the data, statistically significant differences in sugar water and pollen use between treatments were tested with Kruskal–Wallis tests.

### RNA-seq analysis

RNA was extracted from individual larvae following the E.Z.N.A. Total RNA Kit I (Omega Bio-tek) protocol. Larvae were homogenized from frozen in the kit buffer, and subsequently processed following the manufacturer’s instructions. We included a DNase I (Omega Bio-tek) treatment step to degrade remaining genomic DNA. After assessing the RNA quality with an agarose gel (1% w/v), three larval RNA samples were pooled per microcolony, resulting in a final RNA yield of 1 µg per pooled sample. A total of four 0.7 ppb imidacloprid, four 7.0 ppb imidacloprid and four control replicates yielded 12 pooled (three larvae per pool) samples. Pooled RNA samples from a microcolony were treated with poly-A tail selection and sequenced using Illumina technology (HiSeq4000, W.M. Keck Center for Comparative and Functional Genomics, Roy J. Carver Biotechnology Center, University of Illinois Urbana-Champaign), which yielded a total of 386,462,102 single-end reads from the 12 RNA libraries, with an average number of reads of 32,205,175 (minimum–maximum values: 27,142,118–39,048,533). The raw reads are available in the SRA repository (NCBI), with accession IDs SRR20446816-SRR20446827.

Adapter sequences and bases with low quality (Phred < 28) were trimmed from reads with Trimmomatic v0.38^81^; trimmed reads were aligned to the *B. impatiens* genome v2.2^82^ with STAR 2.7^83^, and read counts summarized from the genome's gene features with *htseq-count*^84^ using the "union" method. Alignment of the reads to the *B. impatiens* genome resulted in 96.48% (96.00–96.90%) of reads aligned to the genome, with 84.57% (82.00–87.00%) of them uniquely aligned to a gene feature.

*DESeq2*^85^ was used to normalize the expression values between all the samples, using the *DESeq2* median of ratios method, and under an experimental design where the treatment was included as a three level factor (control, 0.7 ppb imidacloprid and 7.0 ppb imidacloprid). Clustering of normalized samples was checked with principal component analysis (PCA), using the expression values of the top 500 most expressed genes. Differential gene expression analysis was performed with *DESeq2*, making pairwise comparisons between treatments of the normalized read counts through a likelihood ratio test (LRT), for a total of three comparisons: control vs. 0.7 ppb imidacloprid, control vs. 7.0 ppb imidacloprid, and 0.7 ppb imidacloprid vs. 7.0 ppb imidacloprid. *P*-values of the LRTs were adjusted with false discovery rate (FDR). A gene was considered as differentially expressed (DEG) when its FDR in a treatment comparison was lower than 0.05. Additionally, the source colony effects were tested by repeating the differential expression analysis using as variable the source colony, with four levels (colonies C01, C02, C03 and C04). Venn diagrams comparing the DEG sets from each comparison were made using the on-line tools from http://www.interactivenn.net/, and Fisher's exact tests, with the *phyper* command in R v3.6.1^86^, were used to test whether the overlapping number of DEGs between different DEG sets was statistically greater than expected.

Using the gene identifiers from the NCBI *B. impatiens* genome repository (accessed on April 10, 2020), gene ontology (GO) terms associated with each gene identifier were extracted from the *B. impatiens* genome annotation file hosted at the Hymenoptera genome database^87^ at “https://elsiklab-data.missouri.edu/data/hgd/HGD-GO-Annotation/gaf/” using the Unix commands *grep*, *cut*, *awk* and *sed*. Only the genes expressed in the experiment were used to establish a GO term universe, and GO term enrichment analysis was performed for up-regulated and down-regulated DEG sets with *topGO*^88^ for the biological process ontology. A GO term was considered enriched if the weighted Fisher’s exact test *p*-value, corrected through FDR, was less than 0.05. The logarithm of fold enrichment (logFE) was calculated by dividing the observed counts of a given GO term by its expected counts, and calculating the logarithm to the base 2. All statistical analyses were performed with R v3.6. 1^86^. Graphics were generated with R v3.6.1 (unless stated otherwise) and further edited when necessary with InkScape v1.1.1.

## Results

### Bumble bees exposed to the high imidacloprid concentration treatment use less pollen

**Figure 1:**
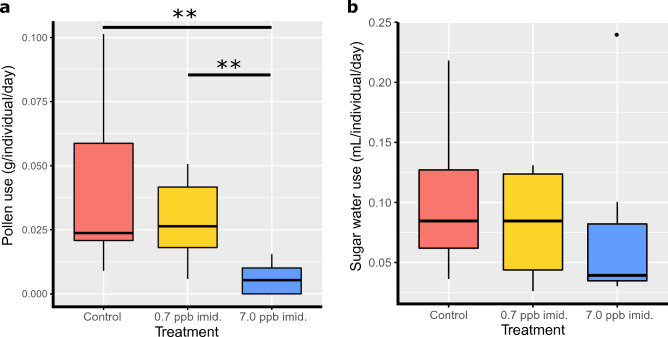
Pollen (**a**) and sugar water (**b**) use by *B. impatiens* microcolonies for control, 0.7 ppb imidacloprid and 7.0 ppb imidacloprid treatments. Horizontal bars between boxes indicate significant differences between treatments, asterisks indicate the Dunn's test significance values associated with the pairwise comparison (**: 0.01 > *p* > 0.001).

Microcolonies in all imidacloprid treatment groups were observed to use pollen and sugar water treatments, but those exposed to 7.0 ppb imidacloprid utilized less pollen than the 0.7 ppb imidacloprid and control treatments (Kruskal–Wallis test χ^2^_2_ = 10.632, *p* = 0.005, Fig. 1a). There was no significant effect of imidacloprid treatment on sugar water consumption (Kruskal–Wallis test χ^2^_2_ = 1.167, *p* = 0.558, Fig. 1b).

### Both high and low concentrations of imidacloprid induced quantitative differences in gene expression

**Figure 2:**
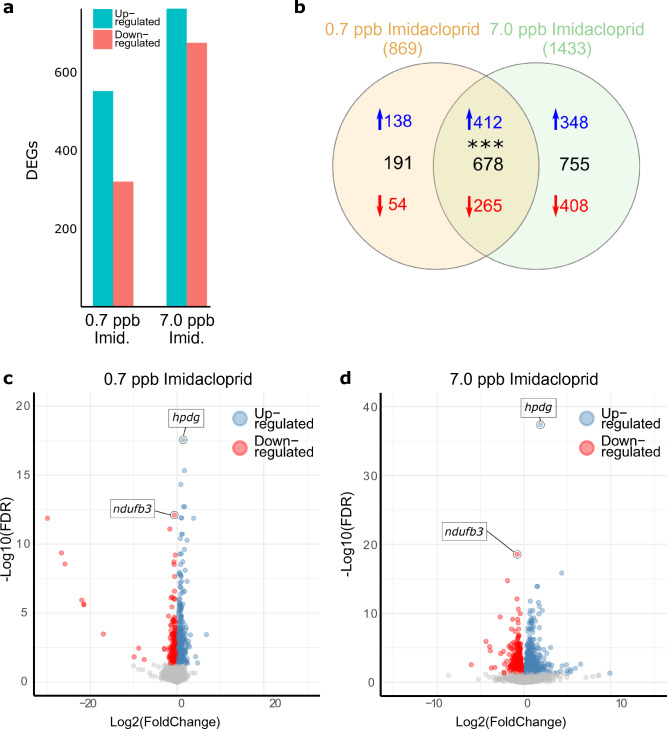
Differential gene expression induced by low and high imidacloprid concentrations. (**a**) Number of up-regulated and down-regulated differentially expressed genes in each treatment. (**b**) Venn diagram with the number of differentially expressed genes (DEGs) associated with the 0.7 and 7.0 ppb imidacloprid concentration treatments when compared to the control treatment. Numbers in parentheses below the imidacloprid concentration treatments are the total number of differentially expressed genes in that group relative to controls. Black numbers indicate the total number of unique or shared DEGs; blue numbers with upward arrows indicate up-regulated genes and red numbers with downward arrows indicate down-regulated genes. Asterisks indicate the hypergeometric test significance (***: *p* < 0.001). Note that up-regulated (blue) and down-regulated (red) DEG values do not exactly match the total DEGs (black) since uncharacterized protein LOC100747518 was down-regulated by the low concentration and up-regulated by the high concentration. Volcano plots for the (**c**) control vs. 0.7 ppb imidacloprid comparison and for the (**d**) control vs. 7.0 ppb imidacloprid comparison: x-axis shows the logarithm to the base 2 of the fold change (FC); y-axis shows the negative logarithm to the base 10 of the false discovery rate (FDR) from DEGs; grey dots represent genes without differential expression (FDR > 0.05); red dots are down-regulated genes and blue dots are up-regulated genes. DEGs with the lowest FDR values are marked with a black circle, pointing to a box including the gene’s short name (*hpdg*: 15-hydroxyprostaglandin dehydrogenase [NAD( +)]-like; *ndufb3*: NADH dehydrogenase [ubiquinone] 1 beta subcomplex subunit 3).

PCA of the 500 top expressed genes, independent of their differential expression (Supplementary Fig. S1), and the heatmap with DEG expression profiles (Supplementary Fig. S2) showed high gene expression variability among the replicates of each treatment, but overall, significant differential gene patterns were detected between the treatments. Relative to controls, we detected 869 differentially expressed genes in the 0.7 ppb imidacloprid treatment (550 up-regulated and 319 down-regulated), and 1,433 (760 up-regulated and 673 down-regulated) DEGs in the 7.0 ppb imidacloprid treatment (Fig. 2a,b, Supplementary Tables S2 and S3). We also found that 982 genes showed source colony effects, 335 of them being DEGs.

### There is a shared set of differentially expressed genes across both concentrations

A shared set of 678 DEGs were affected by both high and low imidacloprid concentrations (Fig. 2b), an overlap that was significantly higher than expected by chance (Fisher’s exact test, *p* < 0.001). All of these DEGs were expressed in the same direction (up- or down-regulated) for both concentrations, except for the uncharacterized protein LOC100747518, which was up-regulated in the high concentration and down-regulated in the low concentration. For both the high and low concentrations, the top up-regulated DEG was a gene homologous to *15-hydroxyprostaglandin dehydrogenase [NAD(* +*)]-like* (LOC100740825, involved in the metabolism of prostaglandins and alcohol dehydrogenase activity in insects, Fig. 2c,d) and the top down-regulated DEG was the *NADH dehydrogenase [ubiquinone] 1 beta subcomplex subunit 3* (LOC100747652, part of the complex that transfers electrons to the respiratory chain in mitochondria, Fig. 2c,d). The top up-regulated overlapping DEGs included those associated with detoxification (three cytochrome P450s (*CYPs*) from the CYP9 family and one cytochrome b5), neural and anatomical development, and hormone regulation (regulators of prostaglandin and juvenile hormone). Among the downregulated overlapping DEGs were genes involved in DNA replication, DNA packaging (histone proteins) and proteolysis (mostly digestive enzymes).

### Different imidacloprid treatments show additional unique, concentration-specific differential expression gene sets

We found 191 DEGs affected exclusively by the low concentration treatment (Fig. 2b). Cytochrome P450s (*CYP6a13* and a *CYP28d1*), venom proteins, cuticle developmental and neural developmental genes were among the up-regulated DEGs. The down-regulated DEGs unique to the low imidacloprid concentration included those associated with cell proliferation and histone proteins. A set of 755 DEGs were associated only with the high concentration treatment (Fig. 2b). The unique upregulated DEGs included cuticle developmental genes, transport proteins, neuropeptide and neurotransmitter receptors, and the detoxification gene *CYP6a14*. DNA replication genes, cell cycle regulatory genes and digestive enzymes were among the down-regulated DEGs specific to the higher imidacloprid concentration treatment. Further, when testing for differential expression between low and high concentration treatments we detected 66 DEGs (Supplementary Table S4): 60 up-regulated in the high concentration compared to the low concentration, and six DEGs up-regulated in the low concentration compared to the high concentration (Supplementary Fig. S3a). Of these DEGs between low and high concentration treatments, 22 of them (Supplementary Fig. S3b) were genes not showing differences in expression when comparing either the low or high concentration treatments against the control.

### GO term enrichment analysis shows that both imidacloprid concentrations down-regulate mitochondrial and DNA replication biological processes

**Figure 3:**
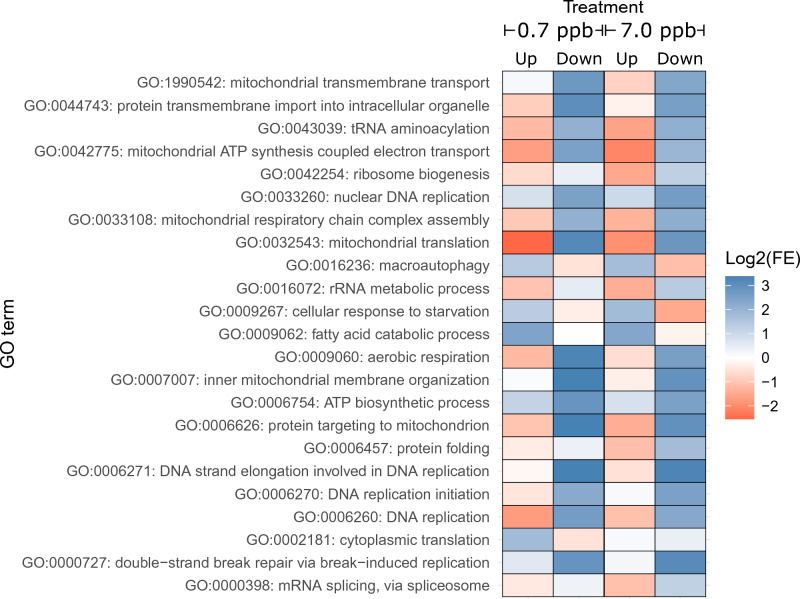
Gene ontology terms enriched in the differentially expressed gene sets. The enriched gene ontology (GO) term names are shown on the Y axis. Tile color indicates values for the logarithm to the base 2 of the fold enrichment of the GO term (i.e., observed count divided by expected count of a specific GO term in the sample). The x-axis shows the up- and down-regulated DEG sets from the low (0.7 ppb) and high (7.0 ppb) imidacloprid treatments. Red tiles represent under-represented GO terms; blue tiles represent enriched GO terms.

Out of the 10,161 GO terms associated with the 9,045 genes from the *B. impatiens* genome expressed in our study, 23 GO terms were enriched in the DEG set (Fig. 3, Supplementary Table S5). The DEGs affected by the low 0.7 ppb imidacloprid concentration showed only cytoplasmic translation (GO:0002181) as enriched for the up-regulated DEGs. Seven GO terms were enriched in the down-regulated DEGs of the low concentration, and were related to mitochondrial activity (5/7) and DNA replication (2/7). Within the DEGs affected by the high 7.0 ppb imidacloprid concentration, the GO terms cellular response to starvation (GO:0009267), macroautophagy (GO:0016236) and fatty acid catabolic process (GO:0009062) were enriched in the up-regulated DEGs. DEGs down-regulated by the high concentration were enriched for 19 GO terms, related to mitochondrial activity (9/19), DNA replication (5/19) and gene expression (5/19). The only enriched GO term in the DEGs from the comparison between the high and the low concentrations was chitin-based cuticle development (GO:0040003), enriched among the up-regulated DEGs.

## Discussion

Our results demonstrate that exposure to both high (7.0 ppb) and low (0.7 ppb) sublethal field-realistic concentrations of imidacloprid in sugar water and pollen resources affect the expression of genes that are important to *B. impatiens* larval development and health. This adds perturbed larval molecular responses to the range of effects already documented for neonicotinoids in bumble bee adults^1^. It appears that the number of genes affected in our study is proportional to the imidacloprid concentration, which has also been found in honey bees^69^. However, we also find that even the lower field-relevant concentrations of imidacloprid directly change the expression of regulatory and other potentially important genes in larvae that could have lasting negative effects on individual and colony health. Follow-up work is required to learn if these molecular effects in larvae could negatively impact larval survival or development, or have negative consequences on adults that have been seen in other studies^55–58,61–63^.

**Figure 4:**
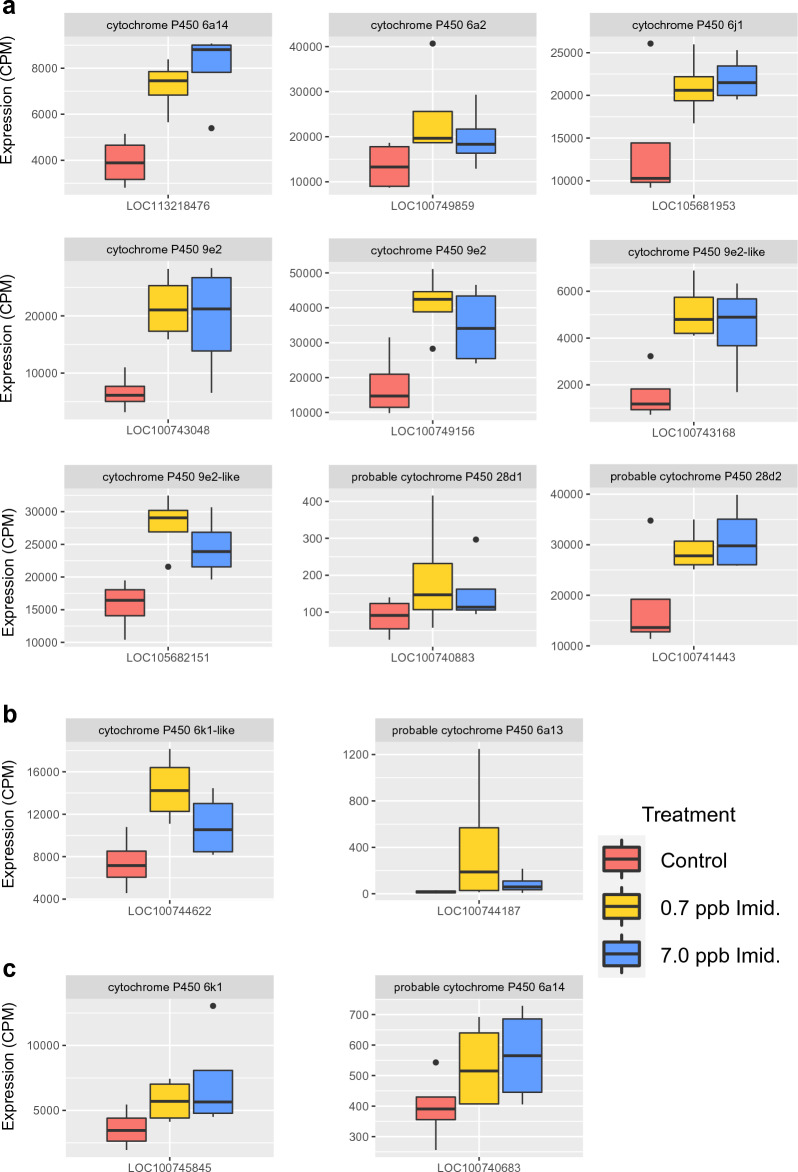
Differentially expressed cytochrome P450s in both 0.7 ppb and 7.0 ppb (**a**), only in the 0.7 ppb treatment (**b**), or only in the 7.0 ppb imidacloprid treatment (**c**), relative to controls. The boxplots show the expression values of cytochromes P450s (CYPs) in read counts per million mapped reads (CPMs, calculated through *DESeq2*), for control, 0.7 ppb imidacloprid and 7.0 ppb imidacloprid treatments. In each boxplot, the title of the plot shows the name of the CYP, and the x-axis shows the unique gene identification number of the CYP.

Imidacloprid is metabolized by cytochrome P450s (CYP) in insects^89–91^, but some of the resulting metabolites are toxic^92,93^**,** and could have side effects on *B. impatiens* health even after degradation of the initial compound. Accordingly, we found an upregulation of *CYP6* and *CYP9* in both imidacloprid concentration treatments (Fig. 4a). Certain gene copies of *CYP6* have been associated with neonicotinoid detoxification in *Drosophila*^89,94^ and in the brown planthopper *Nilaparvata lugens*^95^, and *CYP9* gene copies are involved in honey bee detoxification of acaricides^96^. There were further unique CYPs up-regulated under the low (Fig. 4b) or high (Fig. 4c) imidacloprid treatments. This could suggest the existence of a concentration-dependent regulation of detoxification for either imidacloprid itself or its metabolites. Detecting CYPs whose expression is sensitive to pesticides exposure in bees could help in tracking down orthologous CYPs in other bee species and shed light on the differences of susceptibility to pesticides from different bee species^97^. Other cytochromes, such as *cyt b5* (which enhances CYP activity^98^) and *cyt c* (involved in cell respiration, apoptosis and detoxification^99^), were down-regulated by imidacloprid exposure. Other elements of the mitochondrial cell respiration pathway in addition to *cyt c* were also down-regulated by both concentrations of imidacloprid, such as *NADH dehydrogenase [ubiquinone] 1 beta subcomplex subunit 3*. This is consistent with the literature showing that imidacloprid disrupts mitochondrial activity in insects^100–102^. The inference from this is that no matter the imidacloprid concentration within the range used here, larvae exposed to this pesticide not only face additional energy investments producing CYPs and investing in costly detoxification processes^103,104^, but also experience constraints on their ability to produce energy.

The effects of imidacloprid exposure have been studied more frequently in adults or on whole colonies of bumble bees, but its effect during larval stages, and consequently for larval development and health, remain understudied. We detected that exposure to imidacloprid influenced the expression of genes involved in cell growth regulation and chromatin modification. Several histone proteins (DNA packaging elements) were down-regulated under both the high and low concentration treatments, with a higher number of histone proteins down-regulated under the high concentration, again suggesting a concentration dependent response. Although histone protein depletion induces cell cycle arrest in yeast^105^, the consequences of histone depletion in insect larval development are not yet characterized. Imidacloprid has been shown to induce arrested pupal ecdysis in various Lepidoptera species^106^ and retarded development in honey bees^107^. The gene expression changes in this study may indicate interference with bumble bee larval development as well. The fact that exposure to the high imidacloprid concentration also down-regulates genes related to DNA-replication and cell growth supports a likely effect on development. Slowed larval development combined with previously demonstrated increased worker mortality could explain reduced colony growth under neonicotinoid exposure^46–49^.

Given that neonicotinoids adversely affect learning and memory in bumble bees^108–110^, it was not surprising to find neural activity-related genes affected by imidacloprid exposure. For instance, the transcription factor *GATA-binding factor C-like* protein (LOC100742684, homologous to a protein essential for development and axon guidance^111^) was up-regulated under both treatment concentrations, as well as circadian rhythm proteins (such as *pigment-dispersing hormone peptides*, *protein quiver* and *retinol-binding protein pinta*). Among the DEGs exclusively up-regulated under the high imidacloprid concentration, we found several nervous system and neuron development genes (such as *neurogenin-1, rho GTPase-activating protein 100F, neurogenic locus notch homolog protein 1-like* and *neurofilament heavy polypeptide-like*) and neuropeptide and neurotransmitter receptors (such as *tachykinin-like peptides receptor 86C, neuropeptide CCHamide-1 receptor-like, glutamate receptor ionotropic kainate 2, 5-hydroxytryptamine receptor* or *pyrokinin-1 receptor*), potentially affecting both activity and development of the larval nervous system. The low imidacloprid concentration also up-regulated some nervous system developmental genes (such as *lachesin* and a paralog of the *GATA-binding factor C-like*, LOC100743387), but a smaller set. Larvae of the Asian honey bee *Apis cerana* exposed to imidacloprid can develop into adults with impaired olfactory learning ability^60^. The altered expression of neuronal activity and development genes during the larval stage in *B. impatiens* could generate similar permanent cognitive problems in adults, but this possibility would require further investigation.

In our study, we employed a coarse measure of resource use to confirm the use of spiked food resources and potentially provide context for gene expression patterns. Significantly reduced pollen use in microcolonies exposed to the high (7.0 ppb) imidacloprid concentration (Fig. 1), could explain why we detected up-regulated DEGs associated to the GO term *cellular response to starvation* (GO:0009267) and down-regulated DEGs with digestive functions in this treatment. These DEGs include *pigment-dispersing hormone peptides* (involved in digestion regulation^112^), *sestrin-1* (associated with starvation response^113^), *pyrokinin-1 receptor* (neural receptor involved in insulin production regulation^114^), *chymotrypsin-1* and *digestive cysteine proteinase 1* (both digestive enzymes). The reduction in pollen use in the high imidacloprid concentration treated microcolonies may have been caused by impaired feeding behavior of exposed nurse workers, as seen in^115^, but we cannot confirm this since we did not directly monitor worker feeding behavior. Honey bee workers exposed to imidacloprid show impaired nursing ability, provoking starvation and developmental delay in larvae^107^. While neglect by attending workers is one possibility, these microcolony pollen use and gene expression results could arise from changes in consumption by larvae themselves. Reduced consumption of pollen provisions following neonicotinoid exposure has been shown for larvae of the solitary bee *Osmia*^59^*.* Independent of the root cause, both increased expression of genes relating to starvation and reduced pollen use of microcolonies when exposed to our high field-relevant imidacloprid concentration, suggest that indirect effects due to altered feeding in addition to direct effects of neonicotinoid exposure could be important in affecting larval physiology and health at this exposure concentration. Our study design is not able to tease apart these direct and indirect effects, which would be an interesting future avenue of study.

We see a clear differentiation between gene expression profiles of larvae from the imidacloprid treatments, with many of the patterns expected given previously documented effects. We validated our data comparing the DEG lists with those from four similar studies^68,70,73,74^ (Supplementary Table S1), and found that the overlap between the lists of DEGs from these four studies was greater than expected by chance (Supplementary Table S6). This suggests that our analysis uncovers important general gene expression responses to imidacloprid exposure in *B. impatiens* larvae. Moreover, thirteen genes were consistently differentially expressed in our study and at least another two studies, such as the detoxification genes *cytochrome P450 6k1* (LOC100745845), *reactive oxygen species modulator 1* (LOC100740166) and *alkaline phosphatase* (LOC100749624), the mitochondrial gene *phosphoenolpyruvate carboxykinase [GTP]* (LOC105680266) and the *venom acid phosphatase Acph-1* (LOC105681197), among others (Supplementary Table S6). This set of DEGs might be part of a general molecular response to neonicotinoids in bumble bees and could be used to identify gene expression signatures of exposure in similar studies or in field scenarios. This may extend beyond neonicotinoids, with some DEGs from our analysis potentially representing general responses to xenobiotics, as similar effects have been seen in response to non-insecticide pesticides^116,117^. Despite the clear overall patterns of gene expression differences in larvae following imidacloprid exposure, we see relatively high expression variation across samples from the same treatment that are from different source colonies (Supplementary Figs. S1, S2). This could mean that some genes that are affected in only a subset of the colonies are not identified as differentially expressed in our analysis, even though they could have important consequences for those individuals. Further within colony replication of treatments, absent in our study, would be required to identify if this is the case, as there are other potential explanations for the inter-sample variation. These include, (i) developmental stage differences in sampled larvae that generates noise in the gene expression background, (ii) irregular larval feeding by nurses that induces differences in gene expression between sampled larvae, or (iii) real differences of response to a treatment by larvae from different colonies due to genetic variation.

Overall, our study reveals a molecular basis for a potential detrimental impact of neonicotinoid exposure on larval health, the exact nature of which may vary depending on exposure concentration. We demonstrate that low and high field-realistic sublethal concentrations of imidacloprid, differing by an order of magnitude, trigger molecular responses in *B. impatiens* larvae that include genes involved in key biological processes, including detoxification, neural processes, and larval development. While a core set of differentially expressed genes is shared across the two imidacloprid exposure treatments, each treatment stimulates its own unique differentially expressed gene set, which is larger in the high concentration exposure treatment. Further work is required to pinpoint the exact causes of differential expression profiles between the different exposure concentrations and how these marked molecular responses in larvae affect subsequent health outcomes as they develop into adults.

## Supplementary Information

Supplementary Figures.

Supplementary Table S1.

Supplementary Table S2.

Supplementary Table S3.

Supplementary Table S4.

Supplementary Table S5.

Supplementary Table S6.

## Data Availability

Illumina reads are available at the SRA repository (NCBI), under the BioProject PRJNA861317 (https://www.ncbi.nlm.nih.gov/bioproject/PRJNA861317), with accession numbers from SRR20446816 to SRR20446827. Additional datasets generated and/or analyzed during the current study are available in this published article and its supplementary information files.

## Code availability

Scripts used for data analysis are available from R.M.B. by request.
