# Supplementary material for: Gene expression in bumble bee larvae differs qualitatively between high and low concentration imidacloprid exposure levels
Source: Sci Rep. 2023 Jun 9;13:9415. doi: 10.1038/s41598-023-36232-y (PMC10256756; doi:10.1038/s41598-023-36232-y)
Supplement: Supplementary file 1 — Supplementary Figures. [file 41598_2023_36232_MOESM1_ESM.docx]

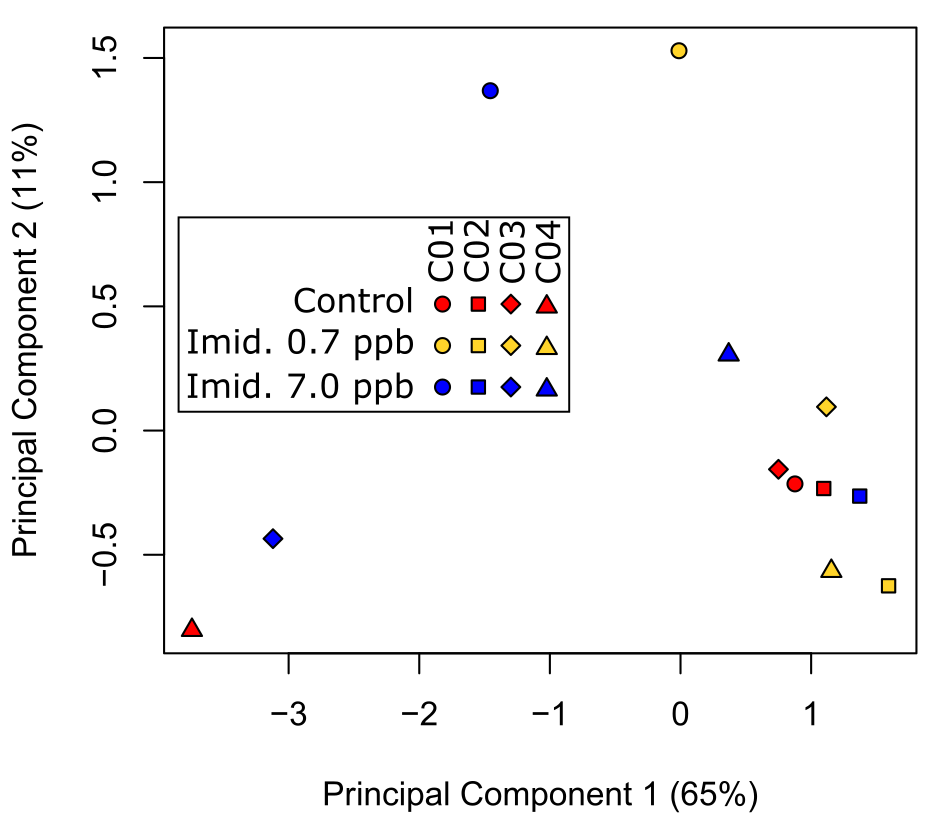


**Supplementary Figure S1.** Principal component analysis of the 500 top expressed genes. Biplot with principal components 1 (x-axis) and 2 (y-axis) with the percentage of variance explained by each in parentheses. The legend indicates the treatment (color) and colony of origin (shape) for each sample pool.


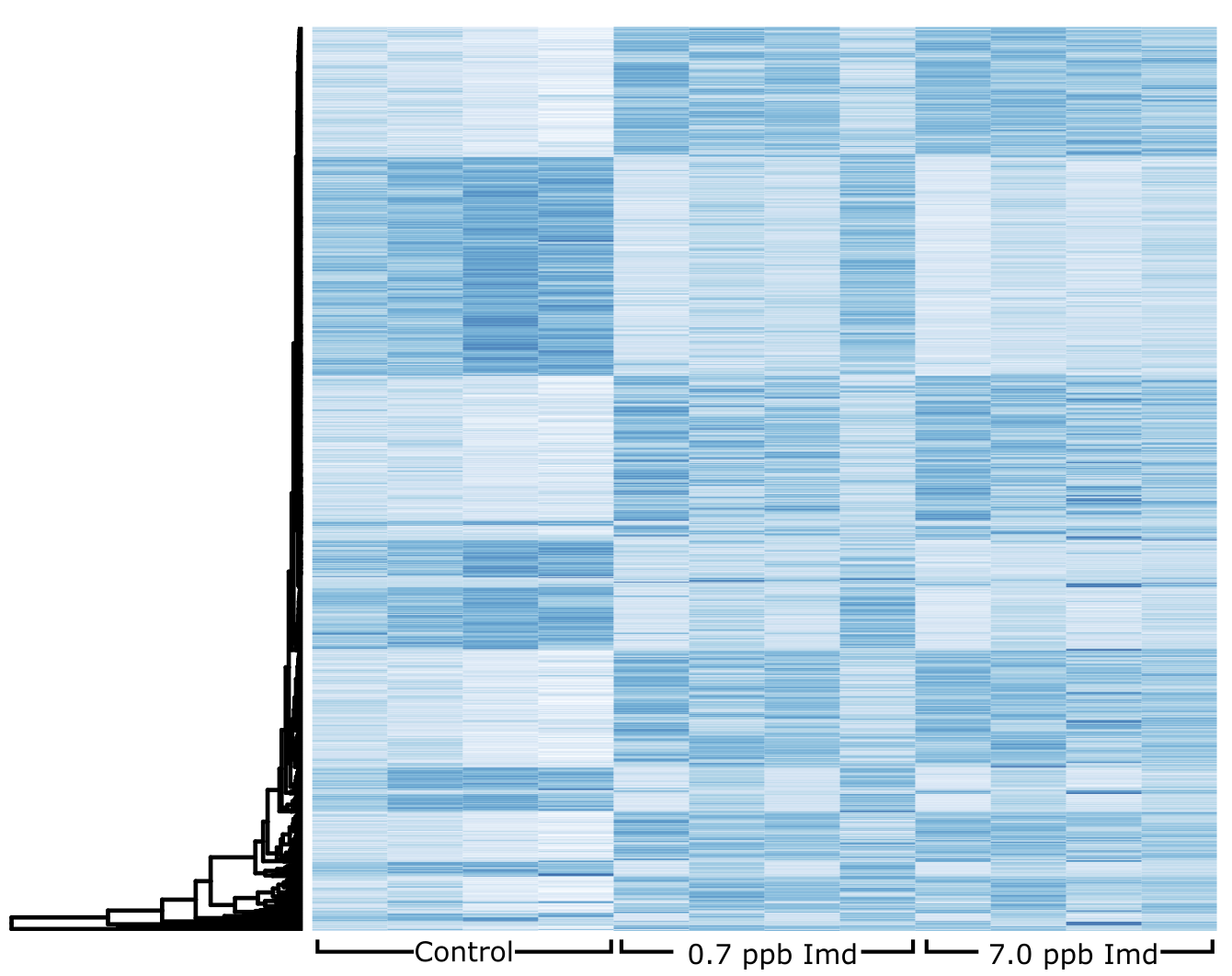


**Supplementary Figure S2.** Expression levels of differentially expressed genes per sample pool. Each column represents a sample, which are grouped by treatment. Treatments are grouped in four columns, each representing a sample pool from a single colony (the order of the colonies for all the treatments is C01, C02, C03 and C04). Each row represents a differentially expressed gene, and each tile represents the counts per million reads (CPM) for a gene in a specific sample. Light blue tiles indicate low CPM values, dark blue tiles indicate high CPM values. The dendrogram on the left side shows the hierarchical clustering of the standardized CPM values.


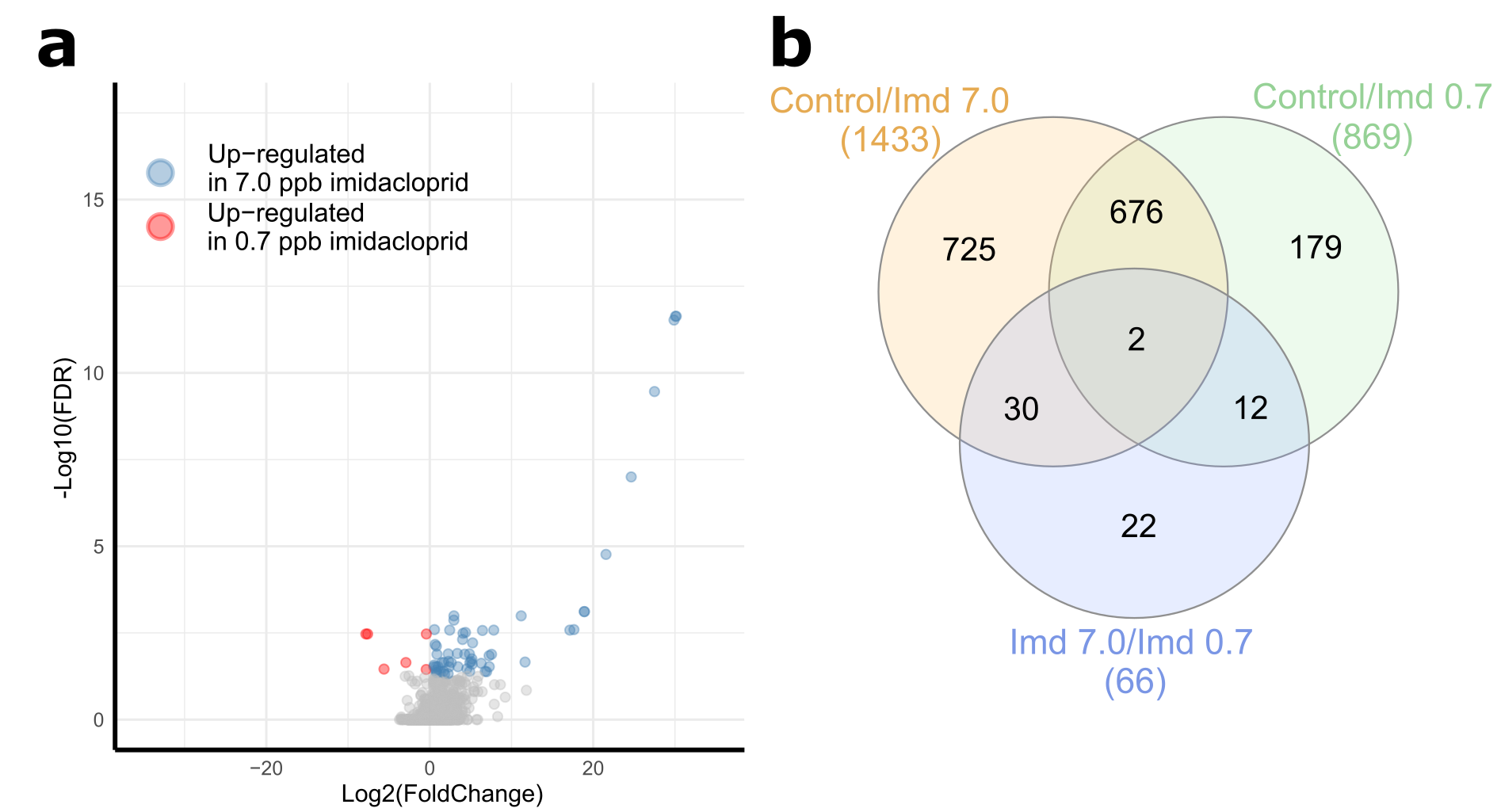


**Supplementary Figure S3.** Results from the comparison between 0.7 ppb imidacloprid and 7.0 ppb imidacloprid treatments. (**a**) Volcano plot for the 0.7 ppb imidacloprid vs 7.0 ppb imidacloprid comparison. X-axis shows the logarithm to the base 2 of the fold change (FC). Y-axis shows the negative logarithm to the base 10 of the false discovery rate (FDR). (**b**) Venn diagram with the number of differentially expressed genes (DEGs) from each set.
